# Cisplatin resistance in gastric cancer cells is involved with GPR30‐mediated epithelial‐mesenchymal transition

**DOI:** 10.1111/jcmm.15055

**Published:** 2020-02-12

**Authors:** Xiaofeng Wang, Zhiyuan Xu, Jiancheng Sun, Hang Lv, Yiping Wang, Yixiu Ni, Shangqi Chen, Can Hu, Lijing Wang, Wei Chen, Xiangdong Cheng

**Affiliations:** ^1^ First Clinical Medical College Zhejiang Chinese Medical University Hangzhou China; ^2^ Department of Abdominal Surgery Zhejiang Cancer Hospital Hangzhou China; ^3^ Key Laboratory of Integrated Traditional Chinese and Western Medicine for Diagnosis and Treatment of Digestive System Tumor Hangzhou China; ^4^ Department of Ultrasonics Zhejiang Cancer Hospital Hangzhou China; ^5^ Cancer Institute of Integrated traditional Chinese and Western Medicine Key Laboratory of Cancer Prevention and Therapy Combining Traditional Chinese and Western Medicine Zhejiang Academy of Traditional Chinese Medicine Hangzhou China

**Keywords:** cisplatin, epithelial‐mesenchymal transition (EMT), G1, G15, gastric cancer, GPR30, resistance

## Abstract

Cisplatin is the major chemotherapeutic drug in gastric cancer, particularly in treating advanced gastric cancer. Tumour cells often develop resistance to chemotherapeutic drugs, which seriously affects the efficacy of chemotherapy. GPR30 is a novel oestrogen receptor that is involved in the invasion, metastasis and drug resistance of many tumours. Targeting GPR30 has been shown to increase the drug sensitivity of breast cancer cells. However, few studies have investigated the role of GPR30 in gastric cancer. Epithelial‐mesenchymal transition (EMT) has been shown to be associated with the development of chemotherapeutic drug resistance. In this study, we demonstrated that GPR30 is involved in cisplatin resistance by promoting EMT in gastric cancer. GPR30 knockdown resulted in increased sensitivity of different gastric cancer (GC) cells to cisplatin and alterations in the epithelial/mesenchymal markers. Furthermore, G15 significantly enhanced the cisplatin sensitivity of GC cells while G1 inhibited this phenomenon. In addition, EMT occurred when AGS and BGC‐823 were treated with cisplatin. Down‐regulation of GPR30 with G15 inhibited this transformation, while G1 promoted it. Taken together, these results revealed the role of GPR30 in the formation of cisplatin resistance, suggesting that targeting GPR30 signalling may be a potential strategy for improving the efficacy of chemotherapy in gastric cancer.

## 1 INTRODUCTION

Gastric cancer (GC) is the fifth most common cancer in the world and the third leading cause of cancer deaths. It accounts for 6.8% of the total cancer incidence and 8.8% of the total mortality rate worldwide, thus posing a serious threat to human health.^1^, ^2^ Due to the lack of obvious clinical symptoms in the early stages of gastric cancer, patients with GC are usually diagnosed only at the advanced stage, resulting in a poor prognosis. Although GC treatment includes surgery, chemotherapy and progress has been made in targeted therapy in recent years, the efficacy has not yet reached expectations.^3^ Chemotherapy plays a very important role in the treatment of GC, especially advanced GC. Platinum drugs (cisplatin and oxaliplatin) are the first‐line treatment in chemotherapy of GC. Cisplatin‐based treatment has been proved to effectively improve the survival rate of patients with advanced GC.^4^, ^5^ Nevertheless, with the widespread use of chemotherapeutic drugs, tumour cells often develop single or multiple drug resistance (MDR) through various mechanisms, which has become a major obstacle in GC chemotherapy.^6^, ^7^ Although some patients are initially sensitive to chemotherapy, almost all patients with GC will develop resistance and recurrence with the continued use of chemotherapeutic drugs.^8^ Therefore, investigating the potential mechanism of chemoresistance is of clinical significance.

The mechanism of resistance development is very complex, and it involves multiple protein‐coding genes and multiple signalling pathways. Epithelial‐mesenchymal transition (EMT) is one of the most widely studied mechanisms not only in GC^9^ but also in pancreatic cancer,^10^ breast cancer,^11^ and lung cancer.^12^ EMT is a reversible process in which epithelial cells lose polarity and cell‐cell adhesion characteristics and acquire mesenchymal cell characteristics.^13^ EMT‐expressing cancer cells showed decreased expression of epithelial cell markers such as E‐cadherin and ZO‐1, while expression of mesenchymal cell markers such as vimentin and N‐cadherin increased. Although there is increasing evidence that EMT is closely related to the development of drug resistance in gastric cancer cells, its underlying mechanisms have not yet been fully elucidated.^14^, ^15^, ^16^

To date, many studies have investigated the role of oestrogen receptor (ER) in gastric cancer, and the possible mechanisms of these effects and the clinical relevance of dysregulated ER in GC. However, the results of these studies are conflicting and controversial.^17^, ^18^, ^19^ GPR30 is a seven‐transmembrane domain protein that not only structurally differs from classical oestrogen receptors (ERα and ERβ) but also significantly differs in terms of its mechanism and effect.^20^, ^21^ Binding of GPR30 to its ligand is known to cause rapid activation of many intracellular signal transduction pathways such as an increase in adenylate cyclase (cAMP), mobilization of intracellular calcium stores, transactivation of EGFR and activation of downstream signal transduction pathways such as PI3K/AKT and ERK1/2. These signalling pathways play an important role in the proliferation, metastasis and formation of drug resistance of human tumours.^22^, ^23^ G1 (GPR30 agonist) and G15 (GPR30 inhibitor) are quinoline derivatives that are widely used to activate or block GPR30 signalling and are used to explore the potential of GPR30 as a therapeutic target.^24^, ^25^ A recent study showed that targeting GPR30 reduced metastasis and drug resistance in breast cancer.^26^ However, few studies have investigated the effect of targeting GPR30 in gastric cancer. Analysis of the KMPLOT database shows that high GPR30 expression leads to poor prognosis in gastric cancer, implicating the potential role of GPR30 in gastric cancer.^27^ In this study, we aimed to investigate the role of GPR30 in the formation of cisplatin resistance in GC cells and to explore the potential of GPR30 as a therapeutic target for improving the efficacy of chemotherapy in GC.

## 2 MATERIALS AND METHODS

### 2.1 Cell culture and reagents

The GC cell lines AGS, BGC823 were obtained from the Cell Bank of Chinese Academy of Science. All the cells were cultured in RPMI‐1640 medium containing 10% foetal bovine serum (FBS) at 37°C with 5% CO_2_. Foetal bovine serum (FBS) and RPMI‐1640 medium were purchased from Gibco (Gibco). G1, G15 and cisplatin were obtained from Sigma‐Aldrich.

### 2.2 siRNA transfection

According to the manufacturer's instructions, GC cells were seeded into the wells of six‐well plates and cultured until approximately 80% confluence, and they were then transfected with GPR30 siRNA (sc‐60743, Santa Cruz Biotechnology) or negative control siRNA (Invitrogen) combined with Lipofectamine 2000 (Invitrogen). The transfection medium (Opti‐MEM) was replaced with complete medium 8 hours after the transfection, and the cells were cultured for 24 hours. The sequences are as follows:

sc‐60743A:

Sense: 5′‐GGAGUUUCCUGUCUGACAAtt‐3′.

Antisense: 5′‐UUGUCAGACAGGAAACUCCtt‐3′.

sc‐60743B:

Sense: 5′‐CUGUGGCUGACGAAUUUGUtt‐3′.

Antisense: 5′‐ACAAAUUCGUCAGCCACAGtt‐3′.

sc‐60743C:

Sense: 5′‐CGUUCAGCCUUUGUCAAUAtt‐3′.

Antisense: 5′‐UAUUGACAAAGGCUGAACGtt‐3′.

Negative Control:

Sense: 5′‐UUCUCCGAACGUGUCACGUdTdT‐3′.

Antisense: 5′‐ACGUGACACGUUCGGAGAAdTdT‐3′.

### 2.3 CCK‐8 cell viability experiment

Gastric cancer cells were seeded into 96‐well plates at a density of 3 × 10^3^ cells/well and incubated at 37°C for 24 hours. They were then cultured in complete medium containing serially diluted drugs for 48 hours. Finally, 10 µL of CCK‐8 was added to each well (Dojindo), and the plates were placed on a microplate reader (MRX II; Dynex) to evaluate the absorbance at 450 nm after incubation for 2 hours at 37°C.

### 2.4 Western blotting

GC cells were treated with a series of concentrations of drugs for 48 hours. The cells were then lysed in RIPA lysis buffer (Cell Signaling Technology) containing protease inhibitor at 4°C. Protein concentration was determined using a BCA protein kit (Thermo Fisher Scientific). The protein was separated by 10%‐15% sodium dodecyl sulphate‐polyacrylamide gel electrophoresis (SDS‐PAGE), then transferred to polyvinylidene fluoride (PVDF) membranes. The membranes were blocked with 5% skim milk and then incubated with primary antibodies against GPR30 (Abcam), E‐cadherin and vimentin (Cell Signaling Technology) overnight at 4°C. Finally, the membranes were incubated with appropriate secondary antibodies (Cell Signaling Technology) for 2 hours after washing with Tris‐buffered saline with Tween (TBST). The blot was visualized using a chemiluminescence kit (GE Healthcare), and protein expression was determined by a Syngene gel imaging system.

### 2.5 EdU assay

The inhibition rate of cell proliferation was determined using a Click‐iT EdU Imaging Kit (Invitrogen), according to the manufacturer's instructions.

### 2.6 Immunofluorescence

Gastric cancer cells were fixed with 4% formaldehyde for 15 minutes after being treated with drug for 48 hours, washed with phosphate‐buffered saline (PBS) and incubated with 5% BSA for 30 minutes at room temperature. Then, the cells were incubated with primary antibodies against E‐cadherin and vimentin (Abcam) overnight at 4°C. After washing with PBS, the cells were incubated with secondary antibodies (Abcam) for 2 hours. The cells were then incubated with 4,6′‐diamidino‐2‐phenylindole (DAPI; Sigma‐Aldrich) for 2 minutes at room temperature. Finally, the cells were washed with PBS and then observed with an inverted fluorescence microscope (Olympus).

### 2.7 Statistical analysis

Each of the above experiments was performed in triplicate and repeated thrice. Statistical analysis was performed using SPSS 23.0 software. Data were expressed as the means ± SD of three independent experiments. Student's *t* test was used for comparison between the two groups. A *P* value of <.05 was considered to represent a statistically significant difference.

## 3 RESULTS

### 3.1 GPR30 participates in cisplatin resistance in GC cells via EMT

**Figure 1:**
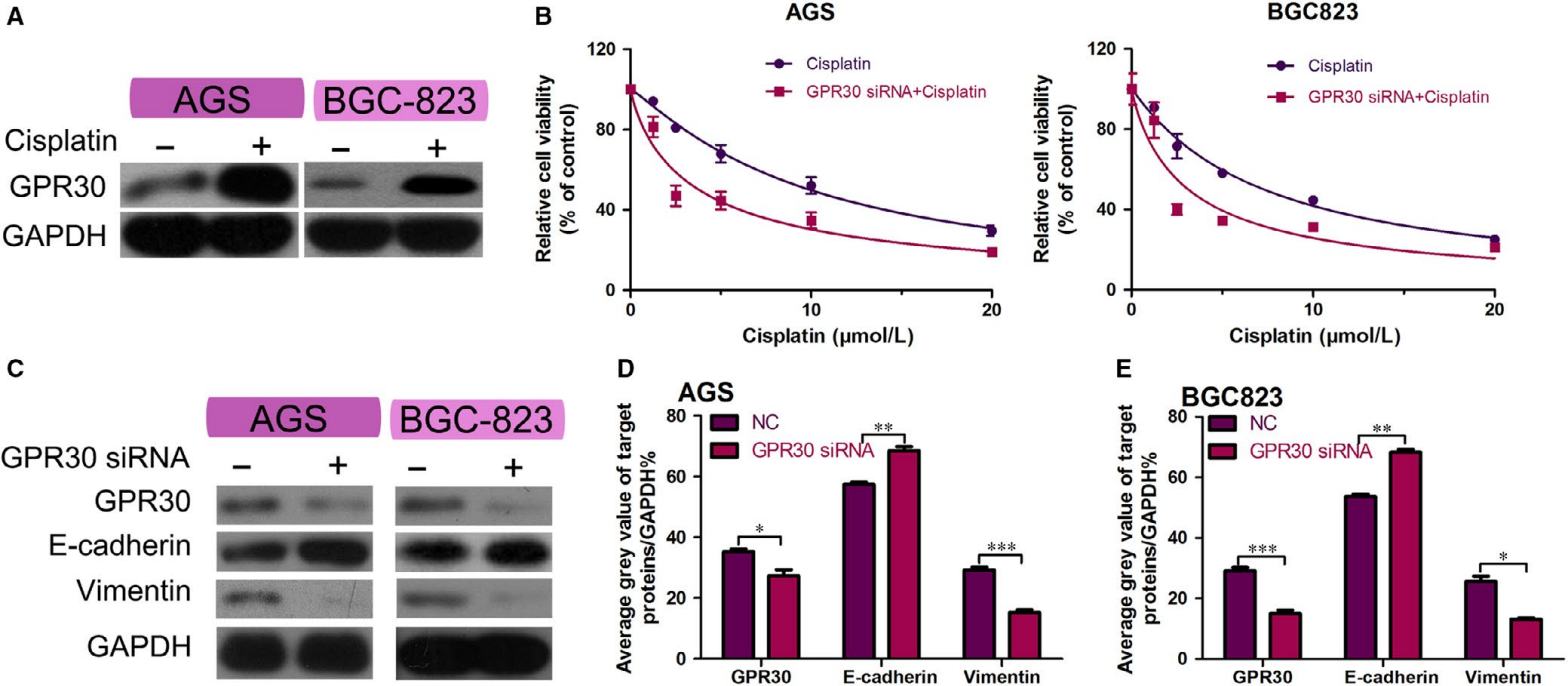
Cisplatin sensitivity and EMT are associated with GPR30 in GC cell lines. A, Western blot analysis of GPR30 in GC cells exposed to cisplatin. B, Viability of AGS, BGC‐823 cells transfected with *GPR30* siRNA or negative control siRNA and treated with a series of concentrations of cisplatin for 48 h, measured using the CCK‐8 assay. C, Western blot analysis of E‐cadherin and vimentin in GC cells transfected with *GPR30* siRNA or negative control siRNA. D,E, *GAPDH* was used as the control to quantify the expression of related proteins in AGS, BGC‐823 cells. *P < .05, **P < .01 and ***P < .001

We found that GPR30 expression displayed increasion in GC cells after cisplatin treatment(Figure 1A). *GPR30* was knocked down in GC cells with *GPR30* siRNA. GC cells were then treated with different concentrations of cisplatin (0‐20 µmol/L), and the CCK‐8 assay was performed to measure the effect of GPR30 on cell proliferation. GPR30 knockdown resulted in increased sensitivity of GC cells to cisplatin to varying degrees (Figure 1B). To investigate the association between GPR30 and EMT, we examined the expression levels of epithelial/mesenchymal markers in GC cells. Results of the Western blot analysis showed that GPR30 knockdown up‐regulated E‐cadherin expression and down‐regulated vimentin expression in GC cells, indicating that GPR30 may be a key factor in regulating EMT (Figure 1C‐E). Twist plasmid assay showed that Twist plasmid enhanced the resistance of GC cells to cisplatin compare with NC group. However, When the cells were cotransfected with Twist plasmid and GPR30 siRNA, the effect of GPR30 knockdown on cisplatin sensitivity disappeared (Figure S1). These data preliminarily demonstrated that GPR30 is involved in the cisplatin resistance of GC cells by promoting EMT.

### 3.2 G15 increases the sensitivity of GC cells to cisplatin

**Figure 2:**
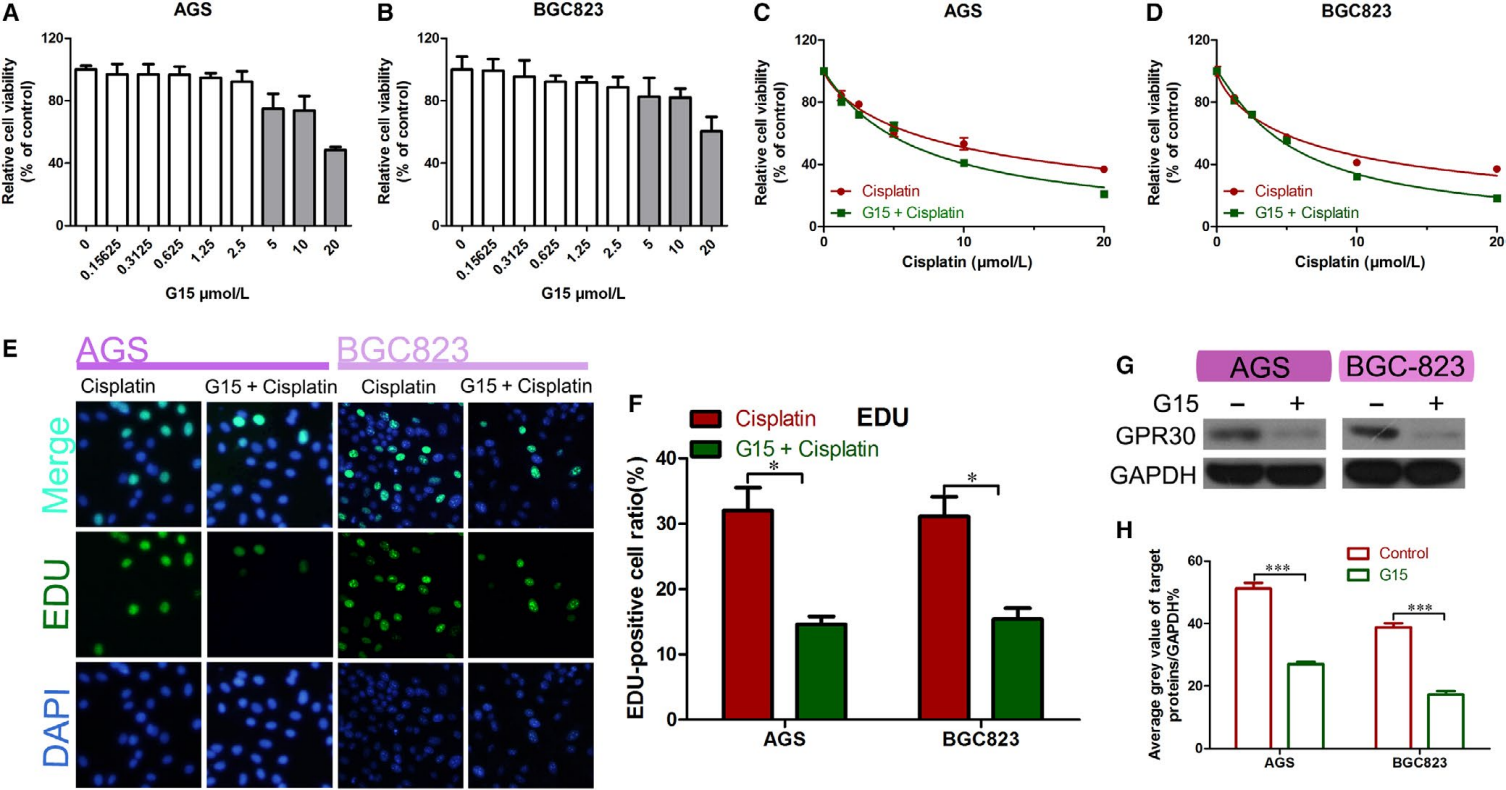
G15 improved cisplatin sensitivity in epithelial GC cells. A,B, Viability of the epithelial GC cell lines AGS and BGC‐823 in a series of G15 concentrations for 48 h as determined using the CCK‐8 assay. C,D, Viability of AGS and BGC‐823 cells treated with cisplatin alone or in combination with G15 for 48 h as determined using the CCK‐8 assay. CI < 1 (combination of G15 and cisplatin) in both AGS and BGC823. E,F, EdU staining assays of AGS and BGC‐823 cells treated with cisplatin alone or in combination with G15 for 48 h. *P < .05. G‐H, Western blot analysis of the efficiency of G15 treatment. ***P < .001

Gastric cancer cells were treated with different concentrations of G15 (0‐20 µmol/L) and their cell viability was measured using the CCK‐8 assay. We selected the highest G15 concentration (2.5 µmol/L) that did not affect cell viability for the next experiment (Figure 2A,B). To investigate the toxic effects of cisplatin and G15 on GC cells, we used the CCK‐8 and EdU staining assays to determine the viability and proliferation of GC cells. G15 was found to significantly increase the cisplatin sensitivity of AGS and BGC‐823 (Figure 2C,D) and inhibit their DNA copies (Figure 2E,F). Western blot analysis was carried out to determine whether G15 effectively inhibited the GPR30 expression. The results indicated that GPR30 expression was significantly inhibited by G15 (Figure 2G,H), suggesting that G15 can increase the sensitivity of GC cells to cisplatin by inhibiting GPR30.

### 3.3 G15 regulates cisplatin‐induced EMT in GC cells

**Figure 3:**
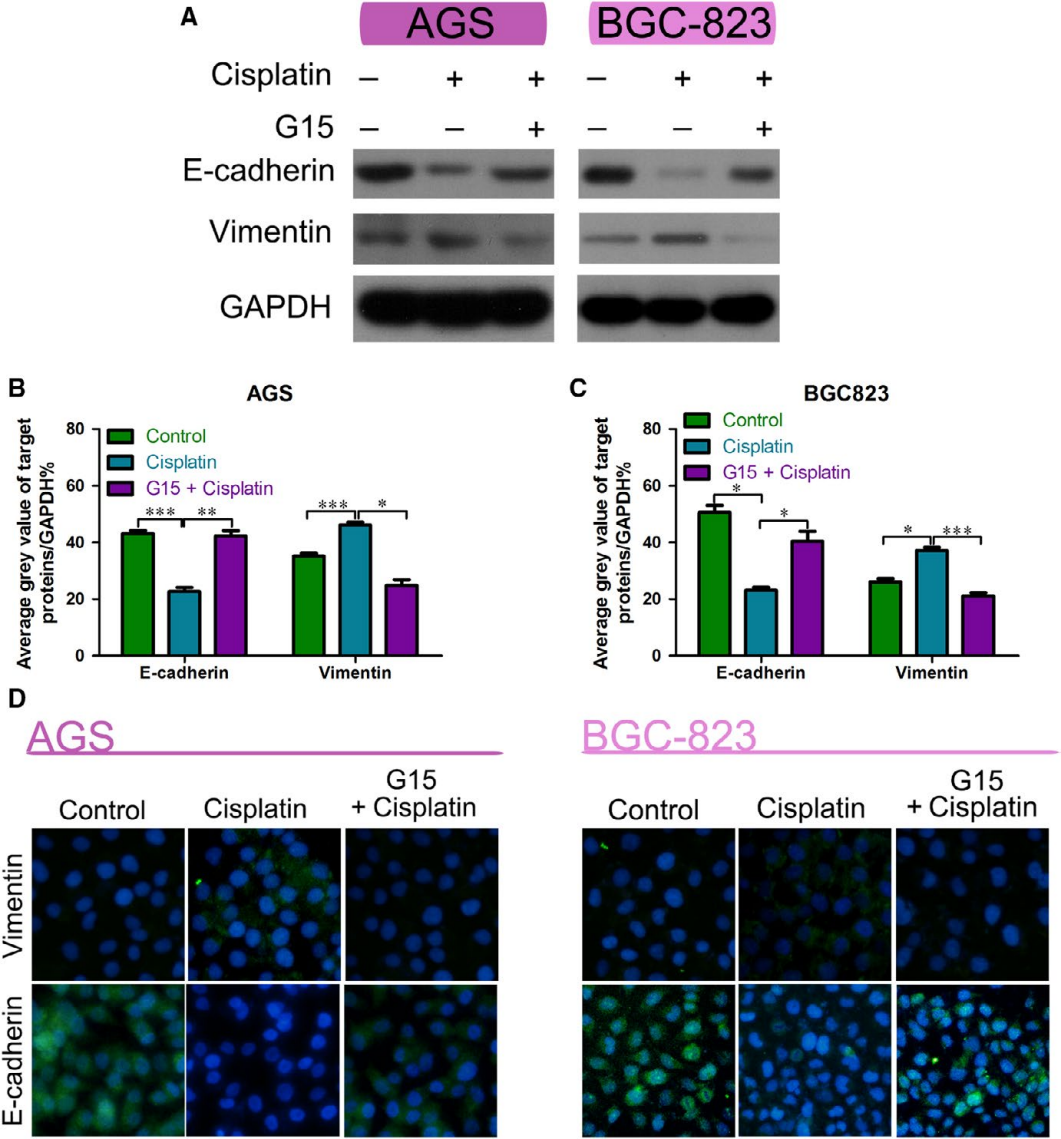
G15 reversed EMT in epithelial GC cells. A‐C, Western blot analysis of E‐cadherin and vimentin in the AGS and BGC‐823 cell lines treated with cisplatin alone or in combination with G15 for 48 h compared with the control. *P < .05, **P < .01 and ***P < .001. D, Immunofluorescence analysis of E‐cadherin and vimentin in the AGS and BGC‐823 cells treated with cisplatin alone or in combination with G15 for 48 h compared with the control

First, to confirm whether cisplatin induces EMT in GC cells, we used cisplatin alone or in combination with G15 on AGS and BGC‐823 cell lines. Western blot analysis showed that E‐cadherin was down‐regulated and vimentin was up‐regulated in cisplatin‐treated GC cells, indicative of EMT. However, G15 used in combination with cisplatin significantly inhibited this change (Figure 3A‐C). Results of the immunofluorescence staining were also consistent with the results of the Western blot analysis (Figure 3D). These data support that G15 reverses the cisplatin‐induced EMT in GC cells.

### 3.4 G15 reverses cisplatin‐induced EMT by inhibiting GPR30

**Figure 4:**
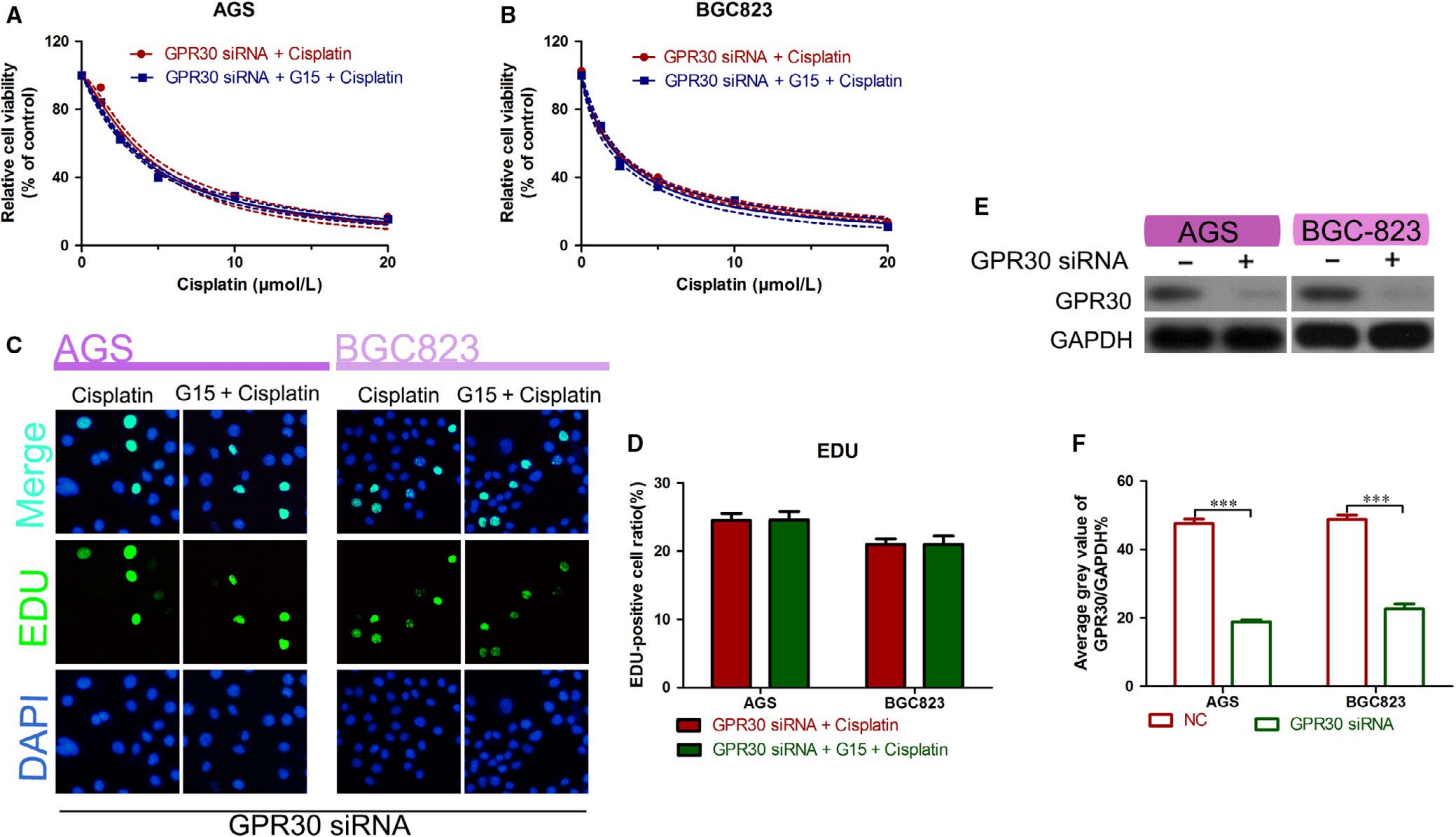
Role of G15 in the inhibition of GPR30. A,B, Viability of AGS and BGC‐823 cell lines transfected with GPR30 siRNA and then treated with cisplatin alone or in combination with G15 for 48 h measured using the CCK‐8 assay. C,D, EdU staining assays of AGS and BGC‐823 cells transfected with GPR30 siRNA and then treated with cisplatin alone or in combination with G15 for 48 h. E,F, Western blot analysis of transfection efficiency of GPR30 siRNA. ***P < .001

To verify that G15 functions via GPR30, we knocked out GPR30 using siRNA and used CCK‐8 to measure the viability of siRNA‐transfected GC cells treated with cisplatin alone or in combination with G15. The results showed that GPR30 knockdown did not significantly affect the G15‐induced cisplatin sensitivity of gastric cancer cells (Figure 4A,B). Results of the EdU incorporation assay also showed consistent results (Figure 4C,D). Western blot analysis showed that GPR30 expression was significantly inhibited when the cells were transfected with GPR30 siRNA (Figure 4E,F). The above results indicate that G15 increases cisplatin sensitivity by inhibiting GPR30, which in turn reverses EMT.

### 3.5 G1 activates GPR30, promoting EMT and leading to cisplatin resistance

**Figure 5:**
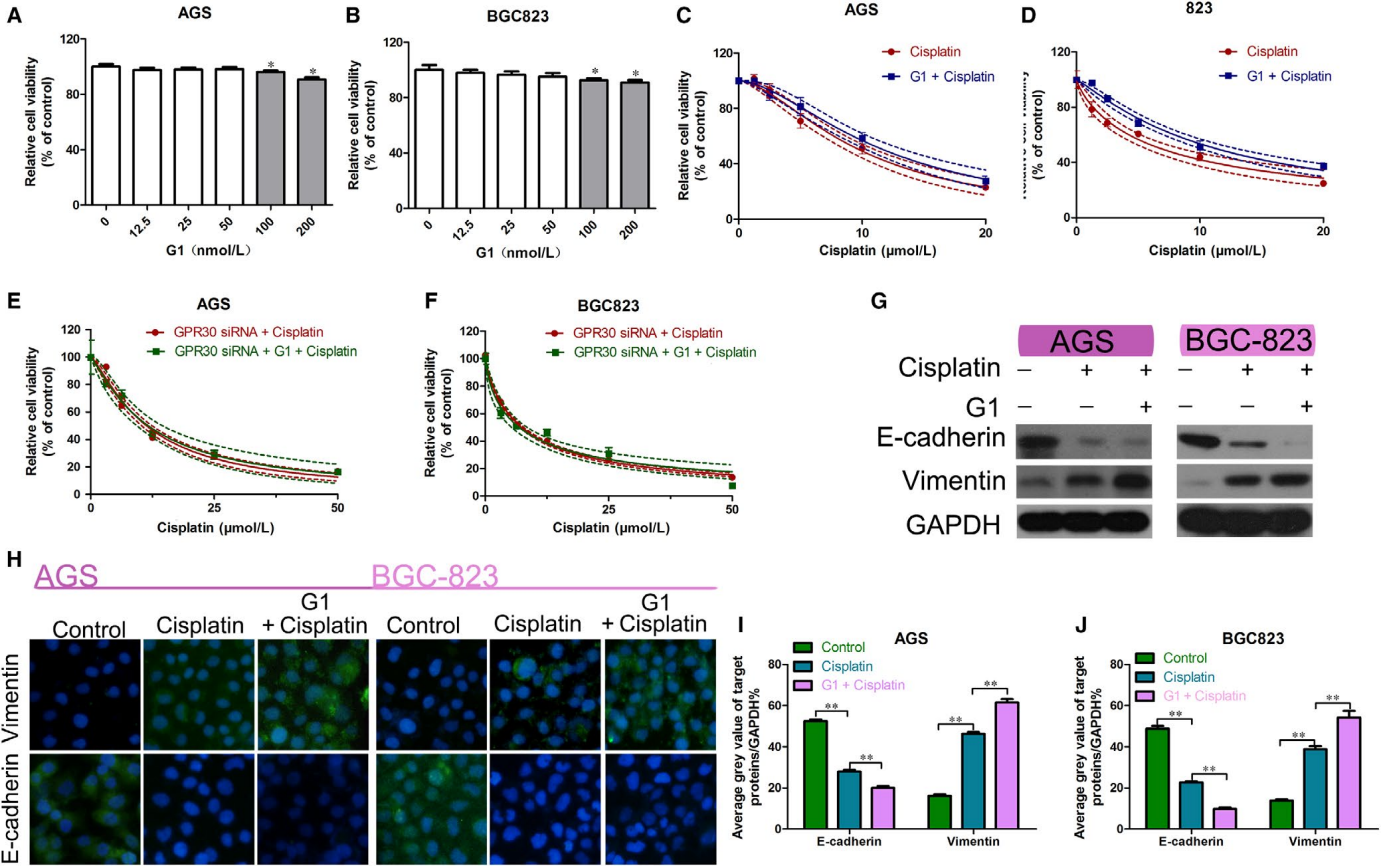
Effect of G1 activation of GPR30 on GC cells. A,B, Viability of the AGS and BGC‐823 cells treated with various concentrations of G1 for 48 h, as measured using the CCK‐8 assay. *P < .05. C,D, Viability of the AGS and BGC‐823 cells treated with cisplatin alone or in combination with G1 for 48 h, as measured using the CCK‐8 assay. E,F, Viability of the AGS and BGC‐823 cells transfected with GPR30 siRNA after treatment with cisplatin alone or in combination with G1 for 48 h, as measured using the CCK‐8 assay. G, Western blot analysis of E‐cadherin and vimentin in AGS and BGC‐823 cells treated with cisplatin alone or in combination with G1 for 48 h compared with the control. H‐J, EdU staining assays of AGS and BGC‐823 cells treated with cisplatin alone or in combination with G1 for 48 h. **P < .01

To further clarify the role of GPR30 in cisplatin‐induced EMT, we used the GPR30 agonist G1 to activate GPR30 to amplify this effect. First, we used the CCK‐8 assay to determine the effect of different concentrations (0‐200 nmol/L) of G1 on the viability of GC cells. Then, we selected the highest concentration (50 nmol/L) of G1 that did not affect cell viability for the subsequent experiment (Figure 5A,B). G1 was found to reduce the sensitivity of GC cells to cisplatin (Figure 5C,D). Next, after siRNA knockdown of GPR30, the GC cells were treated with cisplatin alone or in combination with G1. The results of the CCK‐8 assay showed that G1 did not significantly affect cisplatin sensitivity in these cells (Figure 5E,F). Then, GC cells were used alone or in combination with G1. Western blot analysis showed that cisplatin treatment down‐regulated E‐cadherin expression and up‐regulated vimentin expression in GC cells, and this change was magnified by the addition of G1 (Figure 5G). Immunofluorescence staining also showed consistent results (Figure 5H‐J). These data indicate that G1 promotes EMT by activating GPR30, leading to cisplatin resistance in GC cells.

**Figure 6:**
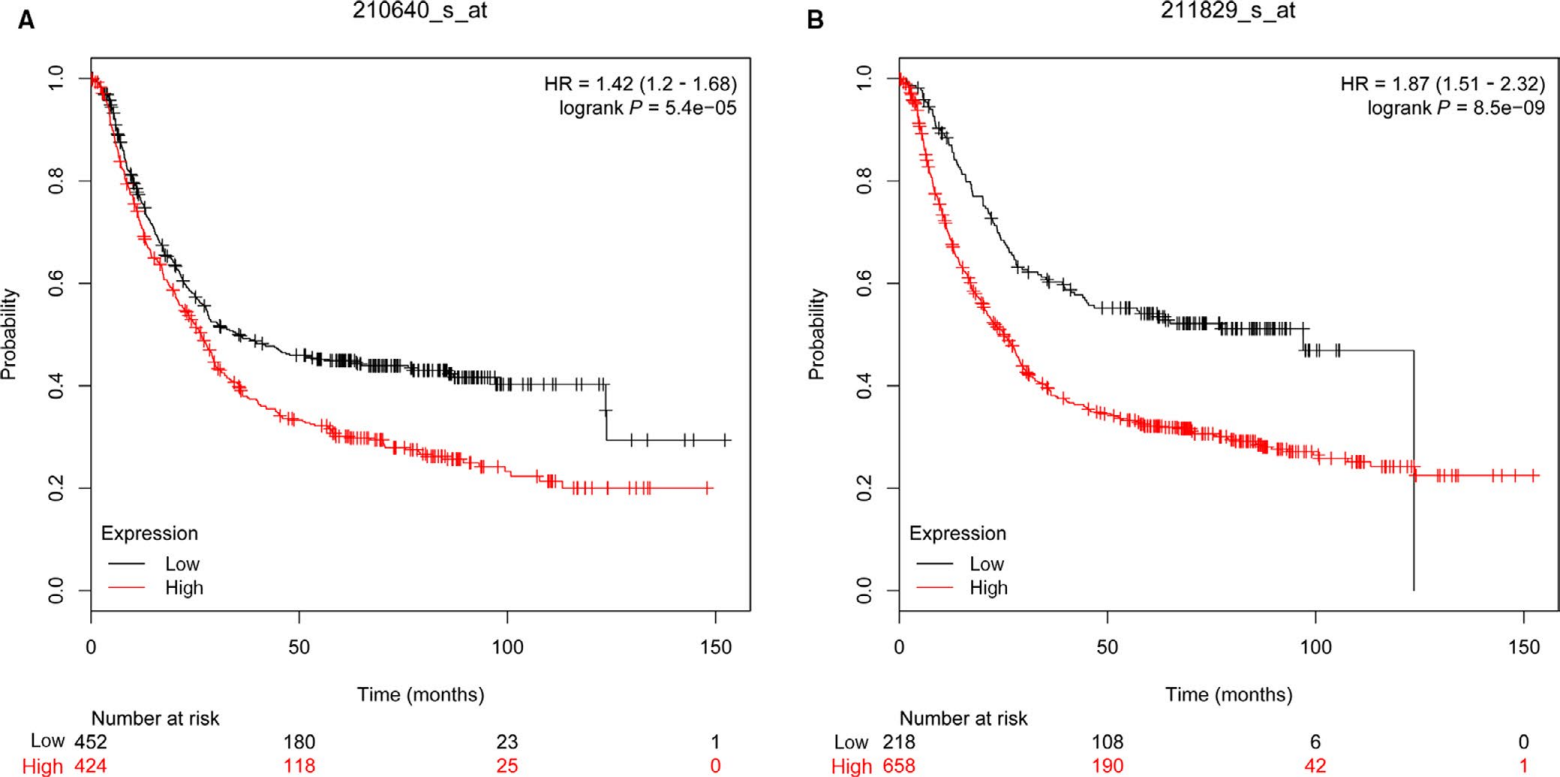
KMPLOT database analysed the expression of GPR30 in GC. A, Affy id/Gene symbol: 210640_s_at; Survival: OS (n = 882); B, Affy id/Gene symbol: 211829_s_at; Survival: OS (n = 882)

In addition, based on the KMPLOT database, we found that a high expression of GPR30 in GC can lead to poor prognosis (Figure 6A,B), suggesting that GPR30 may play a role in tumorigenesis in GC.

## 4 DISCUSSION

Cisplatin has been clinically used as a first‐line chemotherapeutic drug for more than 30 years. Cisplatin alone or in combination with other drugs has been shown to be effective in relieving symptoms, improving the quality of life and improving survival in patients with GC.^28^ Tumour cells develop resistance to chemotherapeutic drugs including cisplatin via a variety of mechanisms, thereby greatly limiting their therapeutic potential. Therefore, it is extremely important to study the mechanisms leading to chemoresistance. Studies have reported that cisplatin is capable of inducing cell apoptosis, which is caused by cross‐linking of intracellular DNA and DNA damage leading to regression of tumours; however, apoptosis can also lead to increased expression of EMT‐inducible factors (such as Twist and Snail) and treatment failure.^29^ These findings indicate that the formation of cisplatin resistance is associated with EMT. Numerous studies have shown that EMT plays an important role in the progression, invasion, metastasis and drug resistance of a variety of cancers including GC.^9^, ^10^, ^11^, ^12^ In this study, E‐cadherin expression was down‐regulated and vimentin expression was up‐regulated in GC cells treated with cisplatin, indicating that GC cells treated with cisplatin experienced EMT‐like changes.

GPR30 is an oestrogen receptor that is independent of the classical oestrogen receptors (ERα and ERβ). Many studies have demonstrated that this novel oestrogen receptor is involved in many cancers through rapid signal transduction events. GPR30 plays a bidirectional role in controlling tumour cell growth, depending on the type of tumour cell 30. Although many researchers have conducted extensive studies on GPR30 and EMT, only few studies have linked them to cisplatin resistance in GC. Our study confirms that GPR30 participates in resistance of GC cells to cisplatin. Specifically, after siRNA knockdown of GPR30 in AGS and BGC‐823 cell lines, these cells showed increased sensitivity to cisplatin and EMT was inhibited. These data suggest that GPR30 is a key factor in the regulation of EMT, determining the resistance of GC cell lines to cisplatin. The findings of this study suggest that GPR30 may promote the involvement of EMT in resistance of GC cells to cisplatin.

G15 and G1 are GPR30‐specific antagonists and agonists, respectively, with no affinity for ERα and ERβ. They have been shown to have potential to treat tumours that express GPR30.^24^, ^25^ Due to the bidirectional nature of GPR30 on tumour growth, G15 limits the progression of non‐small cell lung cancer (NSCLC) by inhibiting GPR30 signalling.^25^ G1 inhibits the growth of prostate tumour cells in vivo and in vitro by activating GPR30 signalling.^30^ These studies suggest that targeting GPR30 may be a potential target for treating cancer more efficiently. Our data revealed that the cytotoxicity of cisplatin is enhanced in AGS and BGC‐823 cells following treatment with G15, whereas treatment with G1 reduced the cytotoxicity of cisplatin towards these cells. After siRNA knockdown of GPR30, neither G1 nor G15 had any effect on cisplatin resistance.

Epithelial‐mesenchymal transition is recognized as one of the main mechanisms leading to chemotherapeutic drug resistance in GC. Many studies have investigated its specific molecular mechanism, although it is still unclear.^31^, ^32^ To explore the association of GPR30 with EMT, we used siRNA to knock down GPR30 in GC cells. GPR30 knockdown resulted in inhibition of EMT in GC cells, suggesting that GPR30 may be an upstream signal of EMT. Furthermore, our data show that G15 reverses cisplatin‐induced EMT in AGS and BGC‐823 cells, while G1 promotes it. These findings reveal that GPR30 is involved in cisplatin resistance in GC by promoting EMT.

In conclusion, the results of this study show that GPR30 plays a non‐negligible role in EMT and cisplatin resistance in GC. Therefore, treatment with targeted GPR30 is a potential strategy to improve the efficacy of chemotherapy in GC. In the future, animal experiments and clinical analyses should be conducted to validate the conclusions of this study.

## CONFLICT OF INTERESTS

The authors declare that they have no competing interests.

## AUTHORS' CONTRIBUTIONS

CXD and CW conceived the research idea; XZY, SJC and LH performed the experiments; WYP, NYX, CSQ, HC and WLJ analysed the data; WXF wrote the manuscript. All authors read and approved the final version of the manuscript.

## Data Availability

All data generated or analysed during this study are included in this published article.
