# Supplementary figures and images for: Cisplatin resistance in gastric cancer cells is involved with GPR30‐mediated epithelial‐mesenchymal transition
Source: J Cell Mol Med. 2020 Feb 12;24(6):3625–33. doi: 10.1111/jcmm.15055 (PMC7131920; doi:10.1111/jcmm.15055)

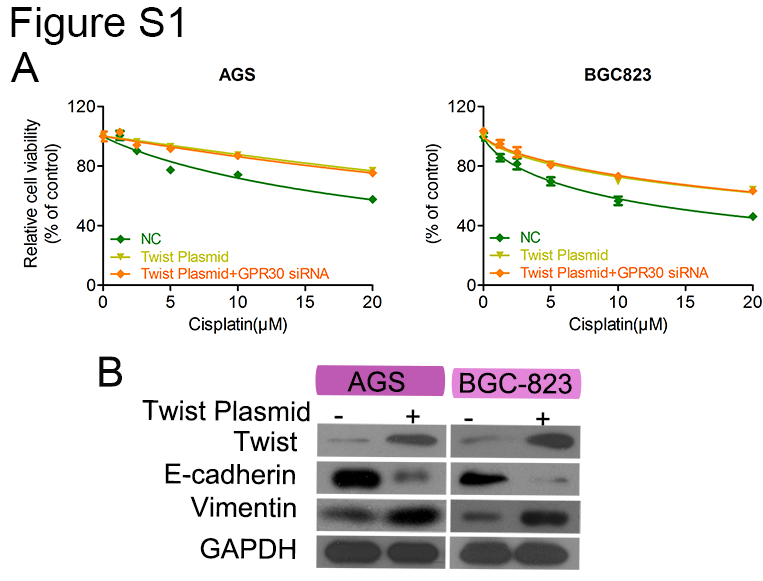

Supplement: Supplementary file 1 [file JCMM-24-3625-s001.tif]
